# Endocrine Disorders in Childhood Cancer Survivors Treated with Haemopoietic Stem Cell Transplantation

**DOI:** 10.3390/children1010048

**Published:** 2014-06-23

**Authors:** Christina Wei, Assunta Albanese

**Affiliations:** 1 St Georges Hospital, St Georges Health Care NHS Trust, Tooting, London SW17 0QT, UK; E-Mail: christina.wei@nhs.net; 2 Royal Marsden Foundation Trust, Downs Road, Sutton, Surrey SM2 5PT, UK

**Keywords:** haemopoietic stem cell transplantation, endocrine disorders, growth hormone deficiency, hypothyroidism, gonadal failure

## Abstract

The increasing number of haemopoietic stem cell transplantations (HSCT) taking place worldwide has offered a cure to many high risk childhood malignancies with an otherwise very poor prognosis. However, HSCT is associated with an increased risk of morbidity and premature death, and patients who have survived the acute complications continue to face lifelong health sequelae as a result of the treatment. Endocrine dysfunction is well described in childhood HSCT survivors treated for malignancies. The endocrine system is highly susceptible to damage from the conditioning therapy, such as, alkylating agents and total body irradiation, which is given prior stem cell infusion. Although not immediately life-threatening, the impact of these abnormalities on the long term health and quality of life in these patients may be considerable. The prevalence, risk factors, clinical approaches to investigations and treatments, as well as the implications of ongoing surveillance of endocrine disorders in childhood HSCT survivors, are discussed in this review.

## 1. Introduction

Haemopoietic stem cell transplantation (HSCT) is the intravenous infusion of multipotent haemopoietic stem cells that are usually derived from the recipient’s own (autologous) or donor’s (allogeneic) bone marrow, peripheral blood, or umbilical cord blood. The commonest indications for HSCT in children are malignancies of the haemopoietic system with poor prognostic factors, or aggressive diseases that have failed to respond to, or relapsed after, conventional chemotherapy. More recently, there has also been an increased number of HSCT in patients with non-malignant haematological conditions such as severe aplastic anaemia, thalassaemia major, and sickle cell disease, as well as, immunological conditions and inborn error of metabolism.

Prior to HSCT, patients undergo myeloablation (or conditioning) with chemotherapy agents such as alkylating drugs, monoclonal antibodies, and in most cases of haematological malignancies, total body irradiation (TBI) as well, in order to eradicate residual malignant cells and promote engraftment by suppressing the recipient’s immune system. TBI is usually delivered in 3–9 fractions instead of a large single fraction in the modern protocols with a total dose ranging from 10–16 Grays (Gy). It has been shown that the delivery of TBI via fractionation results in lower toxicities and gives better outcomes in survivors than a single large dose [1,2]. Patients with diseases involving the central nervous system and/or testes are also given additional radiotherapy to the affected areas. All patients receive standard doses of immunosuppressants to prevent rejection of the donor cells during the first few months post transplantation. Engraftment usually occurs several weeks after stem cell infusion as the expansion of donor cells and their progeny become sufficient to normalise the recipient’s blood counts and reinitiate the immune system.

However, HSCT is associated with a high risk of mortality in the recipients as a result of acute and long-term complications. Acute complications post HSCT include mucositis, sepsis, graft-versus-host disease (GVHD), hepatic veno-occlusive disease, and graft failure due to rejection. Many patients require high doses of antimicrobial and parenteral nutrition support for a number of weeks post HSCT. In addition to standard doses of immunosuppressants, patients with severe and chronic GVHD are treated with higher and/or additional doses of immunosuppressants and corticosteroids. The length of treatment usually depends on the severity and organ involved in the GVHD. Patients who have survived the initial period post HSCT continue to face a high risk of long-term morbidities, such as, second malignancy, cardiovascular diseases, endocrine/metabolic disorders, psychological problems and many others [3,4,5,6,7,8].

This review focuses on endocrine disorders including growth failure, hypogonadism, thyroid problems and abnormal bone mineral density, as well as the increased cardiometabolic risk reported in childhood HSCT survivors.

## 2. Growth Failure and Growth Hormone Deficiency

The reported incidence of growth failure in childhood HSCT survivors ranges from 20%–80% [9]. The mean loss in height in childhood HSCT survivors has been estimated to be approximately 1 height standard deviation score (around 6 cm) compared with both the mean height at the time of HSCT and mean genetic height [10]. Interpretation of growth outcomes from published literature can be challenging due to differences in how growth impairment was defined, heterogeneity in patients’ characteristics and treatment protocols. The commonest endocrine cause of growth failure post HSCT is growth hormone (GH) deficiency, although other factors such as, prolonged steroid therapy due to GVHD, nutritional deficiency, hypothyroidism, gonadal failure and direct irradiation damage to the epiphysis especially at the vertebrae may also play a role.

The somatotropic axis is vulnerable to radiation damage, and GH deficiency is usually the first and commonest pituitary endocrinopathy that manifests itself in HSCT patients conditioned with TBI [11]. Younger children especially those who were transplanted and irradiated under 10 years of age are more susceptible to GH deficiency post irradiation than adults [12], which may occur after a total irradiation dose as low as 10 Gy [2,13,14,15]. However, younger children with GH deficiency respond better to replacement therapy [15]. HSCT survivors who have received single rather than fractionated dose of TBI and/or additional cranial irradiation have a further increased risk of growth failure [10]. The isolated effect of alkylating agents on growth is less clear, but lower rates of growth impairment have been reported in children who are conditioned with cyclophosphamide and busulfan without TBI or cranial irradiation [10,13,16].

The diagnosis of GH deficiency requires GH provocation tests. Discordant GH responses by different provocation tests have been observed in patients with radiation induced GH deficiency [17]. It has been suggested that cranial irradiation causes hypothalamic damage with growth-hormone-releasing-hormone (GHRH) deficiency, as normal GH hormone responses have been observed subjects investigated by tests involving direct stimulation with exogenous GHRH who have demonstrated suboptimal responses by other tests, especially insulin tolerance tests [17]. The insulin tolerance test has been widely accepted as the “gold standard” and regarded as the most sensitive test to identify radiation induced GH deficiency by most paediatric endocrinologists [17]. In clinical practice, GH testing usually takes place when auxological data raise clinical suspicion of GH deficiency, such as, when there is a reduction in height velocity or height measurements are below the parental target range with lack of catch-up growth. Thyroid hormone must be fully replaced in patients with co-existing hypothyroidism before GH testing. Ideally, reversible contributing factors to growth failure, such as nutritional deficiency, should also be addressed before growth hormone testing.

GH treatment should be offered to patients with biochemical evidence of GH deficiency after GH stimulation tests have satisfied the local paediatric cut off and diagnostic criteria for GH deficiency. It has been shown that GH replacement in paediatric HSCT survivors diagnosed with GH deficiency can lead to an increase in height velocity that is adequate to restore a normal growth rate [18]. However, most do not ‘catch up’ adequately to their genetic target height with spinal height particularly affected due to direct irradiation damage to the epiphysis especially at the vertebrae [18].

Patients diagnosed with GH deficiency during childhood should undergo re-assessment once they have reached the end of growth and completed puberty before adult growth hormone replacement is initiated to address adverse long term metabolic health, suboptimal bone mineral density and reduced quality of life associated with GH deficiency.

## 3. Disorder of the Gonadal and Reproductive Function

Hypogonadism, especially hypergonadotropic hypogonadism, is common in survivors of childhood cancer treated with HSCT, and may present as delayed or arrested puberty and infertility [6,11,19]. Although central precocious puberty can also occur in survivors of leukaemia treated with low dose cranial irradiation due to premature activation of the hypothalamic-pituitary-gonadal axis [20], this is uncommon in HSCT survivors treated with TBI.

### Males

In males, germ cells in the testes normally continue to produce sperm during adulthood. However, the germinal epithelium of the seminiferous tubules, responsible for spermatogenesis, is highly sensitive to irradiation damage and chemotherapy toxicity regardless of age [21]. Significant impairment in spermatogenesis has been reported with radiation dose as low as 2–3 Gy [9,22]. Testicular failure with azoospermia has been reported in 48%–85% of male post HSCT, and the chances of recovery are associated with the conditioning regimens [22,23]. For example, the rate of successful pregnancies in female partners of male patients post HSCT treated with cyclophosphamide only was significantly higher compared with those treated with busulphan/cyclophosphamide or TBI [24]. Sperm cryopreservation is currently the best option to preserve future fertility in male adolescents [9,25]. Options of fertility preservation in pre-pubertal boys are still highly experimental.

Leydig cell damage, which leads to reduced testosterone production may also occur in HSCT survivors, especially in those who have required additional local radiation to the testes, and were treated before the onset of puberty [19]. Leydig cells appear more resistant to treatment in postpubertal patients as many demonstrate normal testosterone production despite becoming azoospermic [25]. In prepubertal males, Leydig cell dysfunction commonly presents as delayed (over 14 years of age) or arrested puberty, and is treated by testosterone replacement (from 25–50 mg intramuscular injections 4–6 weekly) with a gradual dose titration to full adult dose (e.g., Sustanon 250 mg intramuscular injections 3 weekly) over 3–4 years. In adult males, testosterone deficiency is associated with an increased risk of adverse metabolic health such as dyslipidaemia, hypertension and hyperinsulinism, which improves with hormone replacement [26]. Male hypogonadism has also been shown to be associated with reduced bone mineral density and osteoporosis [27]. Regular biochemical monitoring of testosterone levels is recommended in post-pubertal male as many patients with hypogonadism are asymptomatic or suffer from non-specific symptoms such as tiredness, poor memory, depression and loss of libido.

### Females

In females, pubertal failure/arrest have been reported in 57% of prepubertal/peripubertal, and ovarian failure in 65%–84% of adult survivors from childhood HSCT [9]. Unlike males, females are born with a finite number of germ cells and the number of primordial follicles reduces with age. In HSCT survivors, this process is accelerated by the effects of treatment, such as, direct radiation damage to the gonads from TBI, and toxicity from chemotherapy, especially alkylating agents. Alkylating agents cause deoxyribonucleic acid (DNA) damage to the underdeveloped oocytes and pre-granulosa cells of the primordial follicles in a dose-dependant fashion [9]. This results in depletion in the number of primordial follicles, cortical fibrosis and subsequent ovarian atrophy. The severity of gonadal toxicity is dependent on the type of alkylating agents used. For example, the use of busulfan during conditioning has shown a greater risk of ovarian failure than cyclophosphamide in female HSCT survivors [28]. The effect of TBI on ovarian function is dependent on the total dose and fractionation of radiation delivered, as well as the age of the patient at treatment [9,29]. It has been shown that up to 50% ovarian follicles in young women can be destroyed by less than 2 Gy of irradiation [30]. Fractionated radiotherapy reduces ovarian damage. Girls treated with 1 Gy in 5 fractions have demonstrated higher chances of spontaneous ovarian recovery than those treated with a single fraction [31]. The risk of ovarian failure increases with the patients’ age [19,32]. Ovarian failure is almost universal in patients who have received HSCT in young adulthood, whereas, those treated before the onset of puberty have a better chance of subsequent ovarian recovery and achievement of spontaneous menarche [19,32], but they remain at a high risk of developing premature menopause. This may be explained by the larger number of non-growing follicles or higher resistance of these follicles to vascular damage and cortical fibrosis induced by chemotherapy in the preadolescent females.

Ovarian function can be monitored by biochemical markers such as follicle-stimulating hormone (FSH) and anti-mullerian hormone (AMH). Raised FSH levels indicate the presence of ovarian failure. However, leukaemia survivors treated with HSCT, who have also received additional high doses of cranial irradiation (>30 Gy), are also at risk of co-existing hypogonadotropic hypogonadism and may not demonstrate raised FSH levels [33]. AMH, produced by granulosa cells of the preantral follicles, correlates with the number of premordial follicles, and has been shown to be a sensitive marker of ovarian function in young adult HSCT survivors of childhood cancer [34]. AMH levels decline with age and are predictors of the age of menopause. However, interpretation is difficult in children and adolescents due to the lack of age and pubertal related reference data.

In addition to ovarian damage, reduced uterine volume, impaired blood flow and absent endometrium have been demonstrated in childhood HSCT survivors [35]. Compared with chemotherapy, TBI has the most deleterious influence on the uterus during conditioning [29]. Uterine volume post HSCT is associated with pubertal status at the time of conditioning, type of conditioning, and the use and dosage of sex hormone therapy [29,35]. The prepubertal uterus is more sensitive to radiation [35]. Although sex hormone replacement enables progression of puberty and achievement of withdrawn bleedings in most HSCT survivors, restoration of normal uterine growth remains inadequate [36].

Recovery of gonadal function in female HSCT survivors occurs in 10%–14% [24]. Patients with residual ovarian function may start and progress through puberty spontaneously. However, longitudinal data have shown that the loss of ovarian function is progressive [34] and premature menopause is common [10]. Ongoing monitoring is therefore required and patients should be advised not to postpone family planning over other life style choices. Less than 3% of HSCT survivors achieve pregnancy, and those who are successful have a high risk of adverse outcomes such as foetal loss, premature delivery and low birth weight [10,24]. Cryopreservation of ovarian tissue for fertility preservation in pre-pubertal females undergoing HSCT is still under development and only available in specialised centres under ethically approved research protocols [9,25]. Significant concerns regarding the potential of reseeding tumour cells following auto- transplantation of ovarian tissues have also been raised [25].

Hormone replacement therapy in females should start at the age of 12–13 years if there are absent signs of puberty and/or raised FSH levels. Oestrogen therapy usually starts at a low dose of oral (e.g., ethinly oestradiol 2 micrograms daily) or transdermal (6.25 micrograms per day twice weekly) preparation, and is increased slowly every 6–12 months, with the aim to attain the full adult replacement dose over 3–4 years. At the onset of uterine breakthrough bleeding or at least 2 years after starting estrogen, progesterone should be added in, either cyclically for 10–14 days of each menstrual cycle (e.g., medroxyprogesterone 5–10 mg or norethisterone 5–10 mg daily) or by switching over to an oral or transdermal combined oestrogen-progesterone contraceptive or hormone replacement preparation.

## 4. Thyroid Disease

Thyroid abnormalities are common in HSCT survivors and can occur as subclinical- compensated hypothyroidism (raised thyroid stimulating hormone (TSH) with normal free T4), overt primary (raised TSH with low free T4) or secondary (inappropriately low or normal or mildly raised TSH with low free T4) hypothyroidism, and autoimmune thyroid disease. The overall reported incidence of thyroid disease in survivors of paediatric HSCT is at around 30%, but ranges from 0%–73% in previous studies, due to variations in the patient cohorts and treatment protocols [9,37,38].

Risk factors for compensated and overt hypothyroidism are often described together in the published literature. The developing thyroid gland is likely to have a greater susceptibility to radiation damage as hypothyroidism is much more common in HSCT survivors treated during childhood than adulthood [39], and has a particularly higher prevalence in children treated under the age of 10 years [9,38,40]. Unlike autoimmune thyroid diseases in the general population, gender does not appear to carry a greater risk of thyroid disease in HSCT survivors [37]. Thyroid dysfunction is also associated with the primary disease and treatment. A greater risk of thyroid disease has been described in HSCT patients with Hodgkin's lymphoma [38], although this may be related to the consequence of the treatment given to these patients rather than the disease itself. The prevalence of hypothyroidism reported is much higher in patients treated with single compared with fractionated TBI, and is much lower in those conditioned with cytotoxics alone [10,38]. In addition to the increased risk of overall thyroid dysfunction, overt hypothyroidism was significantly more common in HSCT survivors treated with single than fractionated TBI (90% *vs*. 14%–15%) [10]. In terms of alkylating agents, busulfan has a greatest risk of hypothyroidism than cyclophosphamide or other alkylating agents [38]. Patients who have received bone marrow or cord blood from unrelated donors are more at risk of developing hypothyroidism than those who have had stem cells from matched sibling donors [37]. The mechanism of this is unclear, but may be immune mediated.

### 4.1. Subclinical Hypothyroidism

Subclinical hypothyroidism, has been described in 7%–31% of childhood HSCT survivors [10,41]. The long term history of irradiation induced thyroid dysfunction and the outcomes of thyroxine replacement in HSCT survivors with compensated hypothyroidism are unknown. The benefit of thyroid hormone replacement in asymptomatic cases of compensated hypothyroidism has been subjected to debate as most cases are asymptomatic, mild and may resolve spontaneously [10]. However, links between the risk of thyroid malignancy and persistently raised TSH have been reported in animal studies, and given the increased risk of second cancer in childhood cancer survivors, treatment with low dose thyroxine to normalise TSH may be justified [10,42]. In clinical practice, HSCT survivors with compensated hypothyroidism should be monitored by 3 to 6 monthly repeat TSH and free T4 levels, and thyroxine replacement should be considered if TSH remains persistently high or continues to rise.

### 4.2. Overt Hypothyroidism

Overt or uncompensated hypothyroidism is usually primary caused by direct damage to the thyroid gland, but can also very rarely be secondary due to irradiation damage to the pituitary gland in patients who have received additional doses of cranial irradiation. Treatment with thyroxine is indicated in all cases of uncompensated primary or secondary hypothyroidism. Thyroid hormone levels should be measured 6–8 weeks after initiation of replacement therapy, and dosage should be adjusted according to thyroid function evaluation every 6–12 months.

### 4.3. Autoimmune Thyroid Disease

Autoimmune thyroid diseases including Hashimotos thyroiditis and Graves’ disease, presumably transferred via donor cells, have also been reported in HSCT cancer survivors [43]. Management of these conditions should be based on the same treatment pathway used in the non HSCT patients.

### 4.4. Thyroid Malignancies

Ongoing surveillance is required in all HSCT survivors as the long term risk of thyroid abnormalities remain and have been reported for up to 28 years post HSCT [38]. The small risk of thyroid tumours in HSCT survivors treated with TBI should also be noted. Saunders *et al*. reported the presence of thyroid malignancies in 13 out of 18 cases of thyroid tumour in a cohort of 791 HSCT survivors from childhood and all underwent total thyroidectomy followed by thyroxine replacement [38]. The risk of thyroid malignancy was not predictable by biochemical testing [38]. Therefore regular neck examination must be included in the patients’ regular follow up, and thyroid imagining by ultrasonography needs be considered in suspected cases.

## 5. Abnormalities of the Hypothalamic–Pituitary–Adrenal Axis

The hypothalamic–pituitary–adrenal axis is more resistant to radiation damage, although HSCT survivors who required prolonged corticosteroid therapy due to GVHD can develop transient steroid-induced adrenal suppression [44]. These patients should be treated with low dose steroid replacement (e.g., hydrocortisone 8–10 mg/m^2^/day) until evidence of adrenal recovery have been demonstrated biochemically by a synacthen stimulation test. There is no evidence in the current medical literature of primary adrenal damage described in HSCT survivors.

## 6. Impaired Bone Mineral Density

The attainment of optimal bone mineral density during childhood, adolescence and early adulthood is an important determinant of long term bone health in order to prevent future risks of osteoporosis and fractures. Reduced total bone mineral density (BMD) with Z scores of <−1 and <−2 have been reported as up to 49% and 21% of childhood HSCT survivors respectively [45,46]. However, adjustments of Z-scores may be required in patients with short stature or delayed puberty. The loss of bone mass is caused by suppression of bone maturation with impaired bone mineralisation due to disturbed calcium and vitamin D homeostasis, as well as, osteoblasts and osteoclasts dysfunction [47].

The aetiology of bone loss after childhood HSCT is multi-factorial and may be associated with the patients’ demographic background (e.g., age, gender, ethnicity), lifestyle, nutritional status and treatment. Patients who have received HSCT at a younger age are at a higher risk of low BMD, suggesting HSCT during childhood interrupts a critical period of bone acquisition with a lasting effect beyond the treatment period [48]. The risk of gender on osteopenia and fractures in HSCT survivors is conflicting [45,48,49].

Treatment specific factors may be directly associated with the oncology treatment itself or secondary to its sequelae. The adverse effects of glucocorticoids on bone health have been well-described in cancer survivors, particularly among those treated with prolonged courses for GVHD [45]. Dexamethasone, which has replaced prednisolone in modern protocols for leukaemia due to its better long term efficacy, is unfortunately associated with greater risks of fractures and reduced BMD [45]. Other cytotoxics have also been shown to cause osteopenia and fractures, such as methotrexate used in the treatment of acute lymphoblastic leukaemia, probably due to its anti-folic acid effects and inhibition of osteoblasts proliferation [50]. TBI and cranial irradiation contribute to the reduction in bone mineral density secondary to hypopituitarism such as growth hormone deficiency and hypogonadism [45]. TBI itself may also cause direct damage to the skeleton leading to damage to the osteoprogenitor cells within the bone marrow. Cytokine storm post HSCT stimulating osteoclasts and increasing bone resorption have been proposed as a treatment-related cause of reduced BMD [51].

The low BMD in some paediatric HSCT survivors may also be related to the lack of mechanical loading from sedentary lifestyle and low body weight of the patients. Lower BMD has been shown in patients who have lower levels of physical activities and longer hours of television or computer screen time [52]. Higher BMD has been described in HSCT survivors with greater body weight and higher fat mass indices [53]. As with individuals in the normal population, nutritional intake is likely to play a role in BMD in HSCT survivors. Lower vitamin D and calcium intake have been identified as risk factors of reduced BMD in young adult survivors of childhood HSCT [48,49].

There is limited data on the cost effectiveness of screening and appropriate interventions of low BMD in survivors post childhood HSCT. It is important to optimise nutritional intake of vitamin D and calcium, and treat HSCT related sequelae associated with reduced BMD, such as, growth hormone deficiency and hypogonadism. Patients should be encouraged to take part in regular weight bearing exercise and aim for a healthy body weight. Bisphosphonate therapy may have a role in severe or symptomatic cases. A short term improvement in BMD in paediatric HSCT survivors has been shown with bisphosphonate therapy which was well-tolerated [54]. However, concerns have been raised that prolonged bisphosphonate therapy may render bones more brittle and impair bone healing in other disease groups [55], and the long term outcome of bisphosphonate therapy in HSCT survivors is currently unknown.

## 7. The Metabolic Syndrome

The metabolic syndrome describes a clustering of abnormalities associated with an increased predisposition to cardiovascular morbidity and mortality later in life. There are various diagnostic criteria of the metabolic syndrome, but the common components usually include central obesity, hypertension, dyslipidaemia and insulin resistance [56,57,58,59]. An increased risk of developing metabolic syndrome, or one or more of its components, has been described in survivors of childhood HSCT, in particular those who were also treated with TBI [60].

The recognition of the increased cardiometabolic risk in childhood HSCT survivors requires careful evaluation as many patients are asymptomatic without obvious clinical features. Obesity with a raised body mass index (BMI), which is the key feature of individuals with the metabolic syndrome in the general population, is usually absent in HSCT survivors despite the presence of adverse metabolic profiles [61,62,63,64,65,66]. This is because the BMI assumes normal stature, body proportions and muscle mass, while childhood HSCT survivors often have shorter stature with a relatively greater loss in spinal height and reduced lean mass [62,66].

Hypertension is common among childhood HSCT survivors [60,67,68,69]. Baker *et al.* have shown that HSCT survivors were 2.06 times more likely to develop hypertension than their siblings [67]. Risk factors for hypertension in HSCT survivors includes TBI, corticosteroids and cyclosporin exposure for GVHD, obesity, diabetes and growth hormone deficiency [68,69].

Childhood HSCT survivors have a high prevalence of dyslipidaemia. Previous studies reported the presence of hypertriglyceridaemia in 13%–39% and reduced HDL in 26%–47% of these survivors [60,70,71]. Abnormal fat metabolism associated with adipocyte dysfunction and altered body fat distribution secondary to the effects of TBI have been speculated as the pathophysiology of dyslipidaemia in HSCT survivors [72,73].

Increased risks of impaired glucose tolerance and diabetes have also been reported in adult survivors of childhood HSCT. The Childhood Cancer Survivor Study in the USA showed an odds ratio of 7.2 in leukaemia survivors treated with TBI compared with sibling controls [74]. Previous studies in glucose homeostasis of HSCT often consist of patients from heterogeneous treatment backgrounds and the prevalence of impaired glucose tolerance and diabetes reported ranges from 3.3%–26% and 0%–17% respectively [61,67,70,74,75,76]. Increased insulin resistance has been described in up to 52% in HSCT survivors [70], but the detailed pathophysiology of abnormal glucose tolerance in HSCT survivors remains controversial and requires ongoing investigations.

Evidence in the cost effectiveness of screening and treatment of cardiometabolic abnormalities in survivors of childhood HSCT is lacking. The recommendations in many current late effects guidelines for childhood cancer survivors are not specific to patients treated with HSCT [77,78]. Future research in this area is needed in order to improve the long term cardiovascular outcomes of these survivors.

## 8. Conclusions

Hypothalamic dysfunction and/or multiple pituitary hormone deficiencies, impaired bone mineral density and adverse cardiometabolic profile are common in survivors of childhood HSCT treated for malignancies, especially if conditioning involves TBI and alkylating agents. The risks and severity are associated with multiple factors such as, gender, primary diagnosis, age at HSCT, total dosage and number of fractions of TBI, type and accumulative dosage of chemotherapy agents, and the length of time since transplantation. Some endocrine abnormalities may be sub-clinical with a long latent period after the completion of treatment. Therefore, regular and life-long monitoring by clinical assessments and appropriate biochemical investigations are important. Treatments should aim at restoring normal physiology, reducing premature mortality and improving quality of life in these survivors.
